# A case of lower respiratory tract infection caused by *Pandoraea sputorum* in a human T-lymphotropic virus type 1 carrier

**DOI:** 10.1016/j.idcr.2025.e02359

**Published:** 2025-09-09

**Authors:** Takahiro Ogasawara, Shinsuke Yamanda, Keito Fukuzawa, Yusuke Yamazaki, Tsuyoshi Doman, Soichiro Minami, Akane Narumi, Shin Saito, Tetsuo Odaka, Kana Ono, Hisashi Shimizu, Jun Sugisaka, Tomoiki Aiba, Sachiko Kawana, Yukihiro Toi, Shunichi Sugawara, Yuichiro Kimura

**Affiliations:** Department of Pulmonary Medicine, Sendai Kousei Hospital, Miyagi, Japan

**Keywords:** *Pandoraea sputorum*, Lower respiratory tract infection, Human T-lymphotropic virus type 1 carrier, Non-cystic fibrosis, Rare pathogen

## Abstract

*Pandoraea sputorum* is a rare, multidrug-resistant, non-fermenting Gram-negative bacillus that has emerged as an opportunistic pathogen, particularly in patients with cystic fibrosis (CF). Reports of infections in individuals without CF remain limited. We report a case of a lower respiratory tract infection caused by *Pandoraea sputorum* in an individual without CF. A 66-year-old Japanese man with a history of right upper lobectomy for large cell carcinoma 10 years ago and human T-lymphotropic virus type 1 (HTLV-1) carrier status presented with recurrent hemoptysis and impaired pulmonary function. Imaging revealed centrilobular nodules and bronchial wall thickening. *Stenotrophomonas maltophilia* was initially isolated from the bronchial washing fluid; however, its susceptibility to imipenem raised concerns about potential misidentification. Subsequently, matrix-assisted laser desorption/ionization time-of-flight mass spectrometry confirmed the pathogen as *Pandoraea sputorum*. The patient was successfully treated with a combination of imipenem/cilastatin, trimethoprim-sulfamethoxazole, and minocycline, resulting in the resolution of symptoms, and radiological and functional improvement were confirmed. This case highlights the challenges of identifying *Pandoraea* species using conventional biochemical methods, suggesting the potential underestimation of this pathogen in individuals without CF. It also suggests that although HTLV-1 carriers are generally considered immunocompetent, they exhibit subtle immune dysregulation that predisposes them to opportunistic infections. Therefore, clinicians should consider opportunistic infections, including those caused by *Pandoraea* species, in individuals without CF who have underlying structural lung disease or potential immune modulation, and utilize advanced diagnostic techniques to ensure accurate identification and appropriate treatment.

## Introduction

The genus *Pandoraea* consists of non-glucose-fermenting Gram-negative rods that were first delineated by Coenye et al. in 2000; it initially comprised five species, including *Pandoraea sputorum* [1]. These bacteria are increasingly recognized as opportunistic pathogens, particularly among patients with CF, from whom they are frequently isolated. They have also been associated with progressive deterioration in pulmonary function and marked inflammatory responses in the respiratory epithelium [2], [3]. Notably, *Pandoraea* species are phenotypically similar to members of the genera *Burkholderia*, *Ralstonia*, and *Stenotrophomonas*, which results in frequent misidentification in clinical microbiology laboratories [1].

With the increasing use of advanced diagnostic technologies, such as matrix-assisted laser desorption/ionization time-of-flight (MALDI-TOF) mass spectrometry and 16S ribosomal RNA (rRNA) gene sequencing, species-level identification of *Pandoraea* has improved, revealing that these organisms are likely underdiagnosed, particularly in non-CF populations [4], [5]. Recent reports have also demonstrated that *Pandoraea* species can cause a wide spectrum of infections, including pneumonia, bacteremia, endocarditis, and soft tissue infections, not only in patients with CF, but also in those without CF, particularly among individuals with immunosuppression or those undergoing intensive care due to critical illness, such as multiple trauma or severe coronavirus disease 2019 (COVID-19) [6], [7], [8], [9], [10].

In terms of geographical distribution, *Pandoraea* species have been sporadically reported worldwide, and there is currently no clear evidence of endemicity in specific regions. The genus includes 28 named species that have been isolated from diverse sources, including respiratory specimens (patients with CF), blood, soil, water, and food samples [11]. These findings suggest that *Pandoraea* species. are environmental organisms with global distribution and opportunistic pathogenic potential in vulnerable hosts.

Human T-cell leukemia virus type 1 (HTLV-1) is a retrovirus that primarily infects CD4-positive T lymphocytes and is estimated to affect 10–20 million people worldwide. Endemic regions include Japan, sub-Saharan Africa, the Caribbean, South America, and Indonesia [13]. Although approximately 1–4 % of HTLV-1-infected individuals develop adult T-cell leukemia, and some may develop HTLV-1-associated myelopathy/tropical spastic paraparesis, the vast majority remain lifelong asymptomatic carriers [12]. However, opportunistic infections have been reported even in asymptomatic carriers, suggesting the presence of subclinical immune dysfunction [12], [13], [14].

Here, we report a case of a lower respiratory tract infection caused by *Pandoraea sputorum* in an individual without CF who is a carrier of HTLV-1.

## Case report

A 66-year-old Japanese man presented with hemoptysis and impaired pulmonary function. His medical history included angina pectoris, hypertension, and dyslipidemia. The patient was taking aspirin, clopidogrel, amlodipine, carvedilol, atorvastatin, and ezetimibe. In 2010, he underwent right upper lobectomy for large cell carcinoma of the lung.

During postoperative surveillance, a granular opacity appeared in the right middle lobe in 2016 and gradually progressed over time. In 2017, bronchoscopy was performed to evaluate a suspected lower respiratory tract infection. *Stenotrophomonas maltophilia* was isolated in small quantities from the bronchial washings and identification was performed using the BD BBL™ CRYSTAL™ system. However, it was not considered the causative pathogen due to the low bacterial load and absence of clinical symptoms. Consequently, the patient was monitored without treatment.

In May 2020, he had recurrent hemoptysis, accompanied by worsening radiologic findings and a decline in pulmonary function. In June 2020, sputum culture revealed a high bacterial load (10^5 CFU/mL) of *Stenotrophomonas maltophilia*, identified using the BD BBL™ CRYSTAL™ system. Repeat bronchoscopy was performed in August 2020 based on these findings. On physical examination, the patient’s temperature, heart rate, blood pressure, and peripheral oxygen saturation were 36.4 °C, 68 beats/min, 111/57 mmHg, and 95 % on room air, respectively. Coarse crackles were auscultated in the right lung field. No cardiac murmurs or other abnormal findings were observed. Laboratory testing revealed mildly elevated C-reactive protein levels, with no anemia or coagulopathy. The anti-glycopeptidolipid-core immunoglobulin A antibody test was positive, while the interferon-gamma release assay (ELISPOT) yielded an indeterminate result due to a positive response in the negative control. Serological tests for connective tissue disease were negative, and both the immunoglobulin levels and lymphocyte proliferation assay were within normal limits, suggesting no overt cellular or humoral immunodeficiency. Additionally, serological testing for HTLV-1 was positive. The patient was referred to a hematologist and was diagnosed as an HTLV-1 carrier without any signs of adult T-cell leukemia or related conditions.

**Fig. 1:**
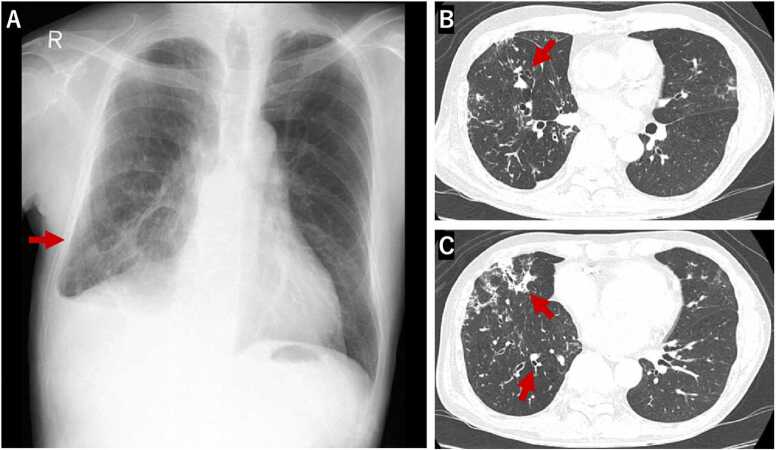
Chest radiograph and computed tomography (CT) scan before diagnosis. (A) The chest radiograph obtained in May 2020, shows ground-glass opacity in the right lower lung field. (B, C) Chest CT scan reveals centrilobular nodules and bronchial wall thickening in the right middle lobe, which raised concern for lower respiratory tract infection.

**Fig. 2:**
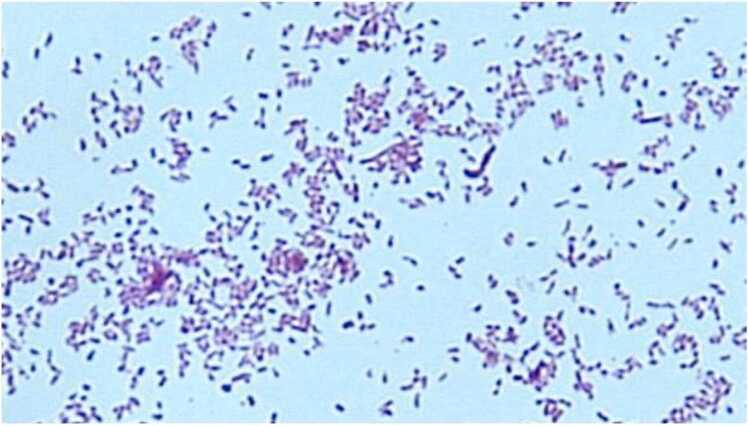
Gram stain of the isolate from bronchial washings obtained via bronchoscopy, showing slender Gram-negative rods.

Chest radiography showed ground-glass opacity in the right lower lung zone, while computed tomography (CT) revealed centrilobular nodules and bronchial wall thickening in the right middle lobe (Fig. 1). Gram-negative rods were identified in the bronchial washings obtained via bronchoscopy (Fig. 2), and initial species identification was conducted using the BD BBL™ CRYSTAL™ system. The antimicrobial susceptibility profile of the isolate from the bronchial washings showed susceptibility to imipenem, which was inconsistent with the typical resistance pattern of *Stenotrophomonas maltophilia* (Table 1). The isolate obtained in 2020 exhibited an antimicrobial susceptibility profile nearly identical to that of the *Stenotrophomonas maltophilia* isolate obtained via bronchoscopy in 2017. Further analysis using MALDI-TOF mass spectrometry identified the organism as *Pandoraea sputorum*. Species-level identification was performed using the VITEK® MS system (bioMérieux, France) with multiple reference libraries. The following libraries were utilized: Filamentous Fungi (468 MSPs, registered 2019–10–09), Mycobacteria Library – bead method (912 MSPs, registered 2018–07–12), BDAL by Bruker Daltonics (7854 MSPs, registered 2019–10–09), and extended microorganism libraries ML01 (114 MSPs), ML02r1 (34 MSPs), and ML03 (37 MSPs), all registered on 2019–07–24. *Pandoraea sputorum* was identified with a confidence value of 99.9 %, supporting the accuracy of the species-level identification. Antimicrobial susceptibility testing showed a profile nearly identical to that of the isolate obtained in 2017, suggesting persistent infection or chronic colonization with the same strain. The susceptibility data were derived from the 2020 isolate, and interpretation was performed using Clinical and Laboratory Standards Institute (CLSI) breakpoints established for *Stenotrophomonas maltophilia*, as no specific breakpoints are available for *Pandoraea sputorum*.

**Table 1:** Antimicrobial susceptibility profile of the isolate obtained from bronchial washing in 2020, interpreted using CLSI breakpoints for *Stenotrophomonas maltophilia*.

Antimicrobial agent	MIC (µg/mL)	Interpretation
Piperacillin	>64	Resistant
Ceftazidime	>32	Resistant
Cefepime	>16	Resistant
Imipenem	1	Susceptible
Meropenem	>16	Resistant
Gentamicin	>8	Resistant
Minocycline	4	Susceptible
Fosfomycin	>128	-
Ciprofloxacin	>2	Resistant
Levofloxacin	>4	Resistant
Trimethoprim-sulfamethoxazole	≤10	Susceptible
Tazobactam/piperacillin	>64	Resistant

*Stenotrophomonas maltophilia* was initially identified in the isolate, although its susceptibility to imipenem was inconsistent with this species’ typical resistance pattern. Further analysis using matrix-assisted laser desorption/ionization time-of-flight mass spectrometry confirmed the organism as *Pandoraea sputorum*.

MIC, Minimum inhibitory concentration

**Fig. 3:**
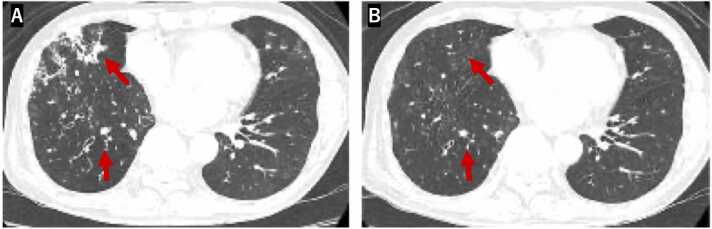
Comparison of chest computed tomography (CT) images before and after treatment. (A) The CT image taken in June 2020 shows centrilobular nodules and bronchial wall thickening in the right middle lobe. (B) The image obtained in December 2020 after 2 months of antimicrobial therapy demonstrates marked resolution of pulmonary infiltrates, in correlation with clinical and functional improvement.

A diagnosis of lower respiratory tract infection caused by *Pandoraea sputorum* was made based on these findings. Antimicrobial therapy was initiated in October 2020 according to the susceptibility profile. The patient received intravenous imipenem/cilastatin at a dose of 0.5 g every 6 h for 7 days, followed by oral trimethoprim-sulfamethoxazole (four tablets daily, in two divided doses) and oral minocycline at a dose of 100 mg/day (50 mg two times daily) for 26 and 14 days, respectively. After 2 months of treatment, hemoptysis resolved, and significant improvements were observed in both pulmonary function and radiologic findings. Vital capacity and forced expiratory volume in 1 s improved from 3.34 to 3.55 L and 2.30–2.45 L, respectively. Chest CT showed marked resolution of the pulmonary infiltrates (Fig. 3).

## Discussion

*Pandoraea sputorum* is an emerging opportunistic pathogen primarily associated with lower respiratory tract infections in patients with CF [15]. However, an increasing number of reports have documented its ability to cause severe infections in those without CF. Ma et al. described a case of sepsis caused by *Pandoraea sputorum* in a 46-year-old female patient without CF following multiple traumatic injuries, demonstrating the organism’s significant virulence and multi-drug resistance, despite treatment with suitable antibiotics [7]. An outbreak in Germany involving 24 critically ill individuals without CF identified *Pandoraea commovens* as the outbreak strain, with common risk factors including invasive procedures and prolonged intensive care unit stays; the isolates exhibited extensive antimicrobial resistance [6]. Lin et al. reported a case of pneumonia caused by *Pandoraea apista* in an individual without CF, underscoring the underestimated virulence of *Pandoraea* species and the diagnostic challenges due to phenotypic similarities with other non-fermenters [3]. Co-infections involving severe acute respiratory syndrome coronavirus 2 and *Pandoraea* species in the respiratory tract have also been reported during the COVID-19 pandemic. Collectively, these findings emphasize the need for heightened clinical awareness and accurate species-level identification to guide effective management of *Pandoraea* infections, even outside the CF population [15]. Among the *Pandoraea* species, *Pandoraea sputorum* has been reported less frequently than *Pandoraea apista* or *Pandoraea pnomenusa*; however, it remains an important pathogen due to its multi-drug resistance and distinct antimicrobial susceptibility patterns [5], [16]. Notably, *Pandoraea* species tend to exhibit resistance to meropenem while retaining susceptibility to imipenem, a pattern believed to result from OXA-type carbapenemases such as OXA-62 and related β-lactamases [16], [17].

Our case highlights several important clinical insights in this context. The patient, a 66-year-old Japanese man, had a history of right upper lobectomy for large cell carcinoma of the lung and was an HTLV-1 carrier. Although HTLV-1 carriers are generally considered immunocompetent, subclinical immune alterations may occur, potentially increasing susceptibility to opportunistic infections.

Previous studies have reported altered T-cell immunity and dysregulated cytokine responses even in asymptomatic HTLV-1 carriers, which may contribute to increased susceptibility to opportunistic infections [14]. Although our patient did not exhibit overt immunodeficiency, the possibility of subclinical immune dysregulation associated with HTLV-1 carrier status cannot be excluded. Furthermore, postoperative structural changes in the lungs may have provided a niche for persistent colonization by *Pandoraea sputorum*. These anatomical changes, which include altered bronchial angles and impaired mucociliary clearance, may mirror the structural airway abnormalities observed in CF, where *Pandoraea* species are known to colonize chronically. Such conditions can facilitate bacterial persistence by limiting effective airway clearance and promoting biofilm formation, as has been reported in CF- and non-CF-related respiratory infections [4]. Of note, the isolate recovered in 2020 demonstrated an antimicrobial susceptibility profile nearly identical to that of an isolate obtained during bronchoscopy in 2017. Although speciation was not confirmed for the 2017 isolate due to the lack of MALDI-TOF analysis, the similarity in susceptibility patterns raises the possibility of long-term colonization by the same species, likely *Pandoraea sputorum*, followed by progression to symptomatic infection. This interpretation remains speculative but aligns with previous findings indicating that chronic colonization by *Pandoraea sputorum* can contribute to pulmonary function deterioration in patients with CF [15] and may extend its relevance to those without CF. The ability of *Pandoraea sputorum* to persist in the airways may be partly attributed to biofilm formation and immune evasion mechanisms, as suggested by *in vitro* studies [2], [15]. These properties may facilitate long-term colonization and delay appropriate antimicrobial intervention in clinical settings.

Although the anti-glycopeptidolipid-core immunoglobulin A antibody test was positive, repeated mycobacterial cultures of sputum were negative, and radiological findings were not suggestive of pulmonary *Mycobacterium avium complex* infection. Similarly, *Mycobacterium tuberculosis* was not suspected based on negative PCR results and negative cultures from bronchial washings. *Nocardia* species and *Aspergillus* species were not detected in sputum cultures. Furthermore, *Pneumocystis jirovecii* and *Aspergillus* species were not clinically suspected due to the absence of characteristic radiographic findings, and specific serological or molecular testing was not performed. The treatment regimen used would also have been appropriate for infections caused by *Nocardia* species or *Pneumocystis jirovecii*. However, the absence of definitive diagnostic testing for these organisms remains a limitation of this case. Nevertheless, given the lack of clinical, radiologic, and microbiological findings suggestive of alternative etiologies, it was considered reasonable to attribute the infection to *Pandoraea sputorum*.

Accurate identification of *Pandoraea* species remains challenging. In our case, the organism was initially misidentified as *Stenotrophomonas maltophilia* both in 2017 and 2020, consistent with previous reports of frequent misidentification among non-fermenting Gram-negative rods [3], [15]. The discrepancy between the organism’s susceptibility to imipenem and the typical resistance profile of *Stenotrophomonas maltophilia* prompted further investigation. Subsequent mass spectrometry-based analysis using the VITEK® MS system (bioMérieux, France) confirmed the organism as Pandoraea sputorum with a confidence level of 99.9 %, supported by updated reference libraries. Although comprehensive accuracy data for MALDI-TOF MS in the identification of *Pandoraea sputorum* remains limited, previous literature has validated its usefulness in species-level identification [15]. While 16S rRNA sequencing may provide definitive identification for rare pathogens, this technique is often reserved for confirmatory purposes due to its higher cost, longer turnaround time, and limited reimbursement under the Japanese national insurance system. In contrast, MALDI-TOF MS is widely covered by insurance and routinely utilized in Japanese clinical laboratories as a rapid and cost-effective primary method of microbial identification. Therefore, in this case, we did not proceed with 16S rRNA sequencing, as the MALDI-TOF results were considered sufficiently reliable for clinical decision-making. This case underscores the importance of considering *Pandoraea* species when encountering unusual antimicrobial susceptibility patterns and highlights the critical role of advanced diagnostic methods, such as mass spectrometry and molecular techniques or 16S rRNA gene sequencing, in achieving accurate identification [15].

Therapeutically, the patient responded well to the combination treatment with imipenem, trimethoprim-sulfamethoxazole, and minocycline. Antimicrobial susceptibility testing was performed using the broth microdilution method, and interpretive criteria were applied based on CLSI breakpoints for *Stenotrophomonas maltophilia*, as no established breakpoints exist for *Pandoraea* species. These interpretations should therefore be made cautiously. This regimen resulted in marked clinical and radiological improvement. Previous reports, including that by Ma et al., have similarly demonstrated the effectiveness of imipenem-based therapy against multi-drug-resistant *Pandoraea sputorum* infections, suggesting that early administration of carbapenems is critical for successful outcomes [7].

To our knowledge, this is the first reported case in Japan of a lower respiratory tract infection caused by *Pandoraea sputorum*. Our case reinforces the emerging recognition of *Pandoraea sputorum* as a clinically relevant pathogen not limited to patients with CF. Structural lung disease and subtle immune modulation, such as HTLV-1 carrier status, may predispose individuals to persistent colonization and eventual infection. Therefore, clinicians should maintain a high index of suspicion for *Pandoraea* infections in individuals without CF who have chronic lung disease or immunologic abnormalities. Accurate species identification and appropriate antimicrobial therapy are also essential for the optimal management of these rare but potentially severe infections.

## Author statement

Shinsuke Yamanda managed the patient and supervised the preparation of the manuscript. Takahiro Ogasawara drafted the manuscript and conducted the literature review. All other authors contributed to clinical interpretation, literature review, or critical revision of the manuscript for important intellectual content. All authors have read and approved the final version of the manuscript and agree to be accountable for all aspects of the work.

## Guarantor

Takahiro Ogasawara.

## CRediT authorship contribution statement

**Tsuyoshi Doman:** Data curation. **Yukihiro Toi:** Supervision. **Yusuke Yamazaki:** Data curation. **Sachiko Kawana:** Supervision. **Keito Fukuzawa:** Data curation. **Tomoiki Aiba:** Supervision. **Shinsuke Yamanda:** Supervision. **Jun Sugisaka:** Supervision. **Takahiro Ogasawara:** Writing – review & editing, Writing – original draft, Data curation, Conceptualization. **Hisashi Shimizu:** Supervision. **Kana Ono:** Supervision. **Tetsuo Odaka:** Supervision. **Shin Saito:** Supervision. **Akane Narumi:** Supervision. **Yuichiro Kimura:** Supervision. **Soichiro Minami:** Data curation. **Shunichi Sugawara:** Supervision.

## Consent

Written informed consent was obtained from the patient for publishing this case report and accompanying images.

## Ethical approval

This study was conducted in accordance with the principles of the Declaration of Helsinki. As this was a retrospective single case report that did not involve any intervention or identifiable personal information, institutional review board (IRB) approval was not required under the policy of our institution.

## Funding

This research did not receive any specific grant from funding agencies in the public, commercial, or not-for-profit sectors.

## Conflict of interest statement

Yukihiro Toi received personal fees from AstraZeneca, Chugai Pharma, Pfizer, Taiho Pharmaceutical, Kyowa Kirin, Bristol-Myers Squibb, Ono Pharmaceutical, and MSD K.K. Shinsuke Yamanda received personal fees from AstraZeneca, Novartis, Sanofi, GSK, and Nippon Boehringer Ingelheim. Shunichi Sugawara received personal fees from AstraZeneca, Chugai Pharma, Pfizer, Taiho Pharmaceutical, Eli Lilly and Company, Novartis, Kyowa Kirin, Bristol-Myers Squibb, Ono Pharmaceutical, MSD K.K., Nippon Boehringer Ingelheim, Takeda, Nippon Kayaku, Merck, Amgen, Thermo Fisher Scientific, Sysmex, and Eisai.
